# Department of Therapeutics

**Published:** 1893-02

**Authors:** David Cerna

**Affiliations:** Galveston


### Abstracts of Current Medical Literature.

### DEPARTMENT OF THERAPEUTICS.

CONDUCTED BY DAVID CERNA, M. D.

### DERMATOL AS AN ANTIDIARRHŒIC REMEDY.

Dermatol, or the sub-gallate of bismuth, has been successfully employed by Colassanti and Dutto (*Berlin Klin. Wochenschrift*, p. 845, No. 34, 1892—*Rev. de Therapeutique Gener. et Thermale*, November 22, 1892), in the treatment of diarrhœa. The remedy was administered internally, in daily doses of from two to six grammes (30 to 90 grains). It was given by itself or associated with the extract of opium. Diarrhœas of tubercular patients were checked by the drug given in amounts of two grammes (30 grains) per day. If the number of the stools exceeded six in the course of the day, dermatol was administered in daily quantities of three grammes (45 grains), divided into six or eight doses

Amelioration was obtained in the course of the twenty-four hours. In less marked cases, one dose of the remedy was sufficient to effect a cure; if a relapse occurred, a second dose produced the desired result. If the number of passages was from ten to fifteen a day, the effect of the drug was less marked, but a cure was generally obtained in from three to four days. According to the authors, the new medicament acts as an astringent and antiseptic, and is useful in the treatment of diarrhœa during the course of typhoid fever, as well as of that occurring during convalescence from the disease. Dermatol produced a rapid cure in ten cases of ulcerative entero-colitis. The same results were obtained in six cases of diarrhœa of malarial origin. The drug was similarly serviceable (especially when combined with opium) in several cases of diarrhœa occurring during convalescence from acute febrile disease. The authors conclude by affirming that dermatol is one of the best and least offensive anti-diarrhœic local medicaments that we have at our command.

---

THE THERAPEUTIC USES OF SALOPHEN.

This drug, a derivative of salol, has been studied therapeutically by Caminer (*Therap. Monatshefte*, p. 544, f. 10, 1892—*Rev. de Therapeutique Gener. et Thermale*, November 22, 1892). In two cases of articular rheumatism, the author prescribed the drug, in doses of one gramme (15 grains), five or six times a day, at intervals of two hours. Under the influence of the drug, the articular pains progressively diminished, the swelling and redness disappeared, and one of the patients regained the use of his limbs in about six days, the other in about ten days. Two months afterwards, no relapse had occurred. The same happy results were observed in ten cases of habitual cephalalgia, that had resisted the internal administration of antipyrin, phenacetine, caffeine, and other medicaments. In these cases, the salophen was given in doses of one gramme (15 grains) every two hours. In most of the instances, relief was obtained after the first dose and after the second or third dose the cephalalgia disappeared. In one patient, however, the good effect was obtained only after the sixth dose. Excellent results were also produced in two cases of supra-orbital neuralgia, in which phenacetine had signally failed. In one of these cases, salophen produced a radical cure. This drug, however, failed in one case of sciatica; but it was effective in many cases of migraine. One patient, suffering from migraine of twenty years' duration, obtained much relief from the use of the medicament. None of the patients treated by the author, exhibited symptoms of poisoning under the administration of salophen.

---

DIGITALIS IN PNEUMONIA.

Belloti (*D. Med. Wochenschrift*, p. 779, No. 34, 1892—*Les Nouveaux Remedes*, November 24, 1892) confirms the favorable action of digitalis, administered in large quantities, in the treatment of fibrinous pneumonia. The medicament is given in daily doses of four grammes (60 grains). Contrary to the statements of Petresco, however, the treatment cannot be considered as an abortive medication.

---

THE ACTION OF JAMBUL.

A new contribution to the action of jambul in the treatment of diabetes, is published by W. Gerlach (*St. Petersburg Med. Wochenschrift*, No. 19, 1892—*Wien Med. Pr.*, No. 26, p. 1158, 1892—*Les Nouveaux Remedes*, November 24, 1892). The writer reports the history of two cases of diabetes, in which the agent under consideration, administered in large doses, did not diminish the glycosuria under a well regulated diet, nor that occurring under the influence of carbo-hydrates given in excess. Is it possible that jambul does not act in the same manner on diabetes of different origin; or were the negative results observed due to the fact that the author only employed the seeds of the syzygium jambolanum? Kobert has seen good effects produced in cases of glycosuria from the use of the extract of the entire fruit of the plant.

---

ANALGEN AND BENZ-ANALGEN.

*Physical and Chemical Properties.*—Analgen is a derivative of chinoline, and was first prepared by G. Vis (*Deutsche Med. Wochenschrift*, No. 44, p. 1005, 1892). It is technically termed *ortho-oxyethyl-anamono-achtyl-amido-chinoline*, its chemical composition being represented by this formula: C H N O. Besides analgen, Vis has obtained, also a derivative of chinoline, a body called *Benz-analgen*, to which the name of *ortho-oxyethyl-anamono-benzoylamido-chinoline* has been given. Analgen occurs in the form of a white powder, has a bitter taste, and is soluble in boiling water, alcohol, and the dilute acids. It has a melting point of 311° F. (155° C.). Benz-analgen is tasteless, and scarcely soluble in water. It is slightly soluble in cold alcohol, but readily so in hot alcohol, as well as in the dilute acids. This new body melts at 406.4° F. (208° C.). Both substances leave no residue on being heated upon platinum wire. Analgen and benz-analgen are dissolved by the gastric juice. Their product appears in the urine in from half an hour to an hour after their ingestion by the stomach. Only one-third of the original amount taken is met with in the urine. This was the general result observed in experiments practiced, within a week. During this time, the amount of ether-sulphuric acids, eliminated by the urine, was not increased; neither was the elimination constant in regard to the other derivatives of the ingested substance. The examination, in three patients, for the detection of chinolinic acid in the urine, only gave negative results. The bodies are broken up in the stomach in this manner: analgen into acetic acid and the amide group, or ortho-oxyethyl-ana-amido-chinoline; benz-analgen into benzoid acid and the same amide combination. The presence in the urine of the ortho-oxyethyl-ana-amido-chinoline is shown by a reddish tint following the ingestion of either analgen or benz-analgen. The drugs acquire anti-putrefactive properties, and that of easily dissolving uric acid.

*Physiological Action.*—The toxicity of the two substances has not been determined as yet. A hypodermic injection of the sulphate of analgen of one gramme (15 grains) to each one of two guinea-pigs, produced convulsions. Dogs tolerated daily doses of three grammes (45 grains), without exhibiting any urinary disturbances. At the autopsy of one of these animals, no renal lesions were discovered.

*Therapeutic Applications.*—Both substances have been employed clinically in the service of Baeumler (*loco cit.*). It was found that the benz-analgen was more efficacious than analgen as a therapeutic agent. The ortho-oxyethyl-anamono-benzoyl-amido-chinoline, administered to a short number of patients, in daily doses of one to two grammes (15 to 30 grains), produced antithermic and antineuralgic effects, similar to those of phenacetine, and without causing untoward symptoms. These results were afterwards confirmed in the service of Loebell (*loco cit.*). Given to phthisical patients, in doses of from one to two grammes (15 to 30 grains), it reduced the temperature to the normal standard. This reduction of temperature was accompanied by profuse sweating, but no other disagreeable effects were observed. On the contrary, the action of the medicament is relatively more prompt than that of other antipyretics. The antineuralgic action of the drug was effective in cephalalgias and essential neuralgias. In one case of arthritis of the hip, the remedy, in daily amounts of three grammes (45 grains), relieved the violent pains, but did not cause these to disappear. It failed in one case of post-diphtheretic neuritis. Satisfactory results were obtained in one case of muscular rheumatism, and in three tabetic patients. In one case of neurasthenia, the relief was slight. Benz-analgen failed in two out of three cases of syphilitic cephalalgia, and in one out of two of myelitis. The drug rendered good service in one case of alcoholic neuritis, and especially in a case of painful right hemicrania, and in another of arthralgia. In all of these latter cases, the action of benz-analgen was efficacious in relieving pain. The results obtained in the service by Jolly, of Berlin, and embodied in the inaugural thesis of P. Krulle (*Inaugur. Dissert.*, Berlin, 1892), are similarly interesting and encouraging. Success, according to Krulle, was observed in eleven out of twenty-four cases treated. Seven of these cases were of cephalagia of traumatic origin; two of them occurred in tabetic patients, in whom the pains were of a lancinating character. In two other cases of tabes, the action of the remedy was uncertain. Loebell refers to other instances coming under the observation of various practitioners, and in which benz-analgen was found of marked service in the treatment of chronic gout, especially in the relief of articular pain. No untoward effects were observed, following the administration of the drug. The dark color of the urine is regarded as insignificant.

*Administration.*—Benz-analgen may be given in daily doses of from 0.5 to 3 grammes (7½ to 45 grains), or even as high as 5 grammes (75 grains).

